# Thrombomodulin Ala455Val Polymorphism and the risk of cerebral infarction in a biracial population: the Stroke Prevention in Young Women Study

**DOI:** 10.1186/1471-2377-4-21

**Published:** 2004-12-01

**Authors:** John W Cole, Stacy C Roberts, Margaret Gallagher, Wayne H Giles, Braxton D Mitchell, Karen K Steinberg, Marcella A Wozniak, Richard F Macko, Laurie J Reinhart, Steven J Kittner

**Affiliations:** 1 Department of Neurology, University of Maryland School of Medicine, Baltimore, Maryland, USA; 2 Epidemiology and Preventive Medicine, University of Maryland School of Medicine, Baltimore, Maryland, USA; 3 Department of Medicine University of Maryland School of Medicine, Baltimore, Maryland, USA; 4 Geriatrics Research, Education, and Clinical Center, Department of Veterans Affairs Medical Center, Baltimore, Maryland, USA; 5 Molecular Biology Branch, National Center for Environmental Health, Centers for Disease Control and Prevention, Atlanta, Georgia, USA; 6 Division of Adult and Community Health, National Center for Chronic Disease Prevention and Health Promotion, Centers for Disease Control and Prevention, Atlanta, Georgia, USA; 7 Coordinating Center for Health Promotion, Centers for Disease Control and Prevention, Atlanta, Georgia, USA

## Abstract

**Background:**

The genes encoding proteins in the thrombomodulin-protein C pathway are promising candidate genes for stroke susceptibility because of their importance in thrombosis regulation and inflammatory response. Several published studies have shown that the Ala455Val thrombomodulin polymorphism is associated with ischemic heart disease, but none has examined the association with stroke. Using data from the Stroke Prevention in Young Women Study, we sought to determine the association between the Ala455Val thrombomodulin polymorphism and the occurrence of ischemic stroke in young women.

**Methods:**

All 59 hospitals in the greater Baltimore-Washington area participated in a population-based case-control study of stroke in young women. We compared 141 cases of first ischemic stroke (44% black) among women 15 to 44 years of age with 210 control subjects (35% black) who were identified by random digit dialing and frequency matched to the cases by age and geographical region of residence. Data on historical risk factors were collected by standardized interview. Genotyping of the thrombomodulin Ala455Val polymorphism was performed by pyrosequencing.

**Results:**

The A allele (frequency = 0.85) was associated with stroke under the recessive model. After adjustment for age, race, cigarette smoking, hypertension, and diabetes, the AA genotype, compared with the AV and VV genotypes combined, was significantly associated with stroke (odds ratio 1.9, 95% CI 1.1–3.3). The AA genotype was more common among black than white control subjects (81% versus 68%) but there was no significant interaction between the risk genotype and race (adjusted odds ratio 2.7 for blacks and 1.6 for whites). A secondary analysis removing all probable (n = 16) and possible (n = 15) cardioembolic strokes demonstrated an increased association (odds ratio 2.2, 95% CI 1.2–4.2).

**Conclusions:**

Among women aged 15 to 44 years, the AA genotype is more prevalent among blacks than whites and is associated with increased risk of early onset ischemic stroke. Removing strokes potentially related to cardioembolic phenomena increased this association. Further studies are needed to determine whether this polymorphism is functionally related to thrombomodulin expression or whether the association is due to population stratification or linkage to a nearby functional polymorphism.

## Background

**Figure 1:**
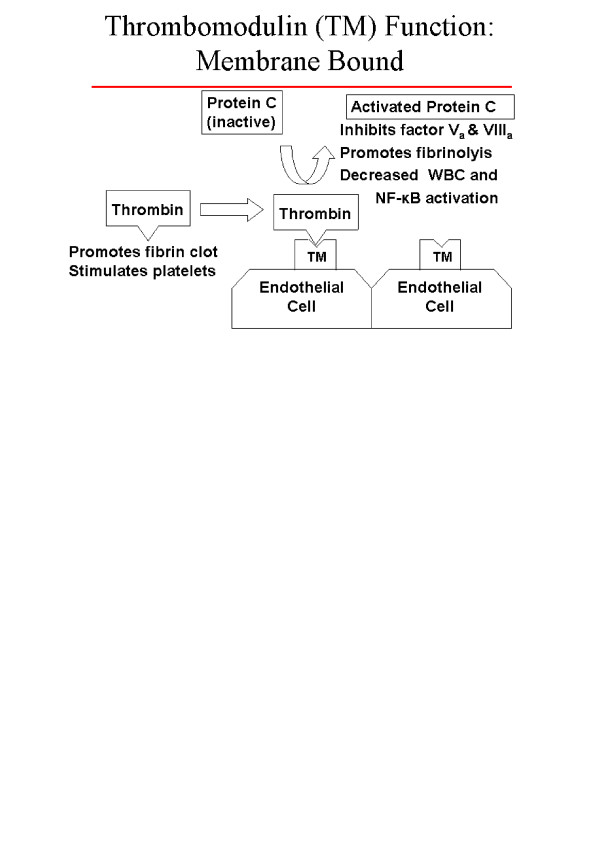
Thrombomodulin / Protein-C relationships and function

Thrombosis is a dynamic balance between factors that promote clot formation, antithrombotic mechanisms, and fibrinolysis. Central to this balance is the thrombomodulin-protein C antithrombotic mechanism. Thrombomodulin forms a 1:1 complex with thrombin on the vascular endothelium, thereby inhibiting the procoagulant actions of thrombin and converting protein C to activated protein C [1]. Activated protein C promotes fibrinolysis, inhibits thrombosis by inactivating clotting factors Va and VIIIa, and reduces inflammation by decreasing white blood cell and nuclear factor kappa-B activation [2-5]. These relationships are demonstrated in Figure 1. Because of the central role that the thrombomodulin-protein C pathway plays in thrombosis regulation and inflammatory response, the genes encoding these pathway proteins are promising candidate genes regarding stroke susceptibility.

**Figure 2:**
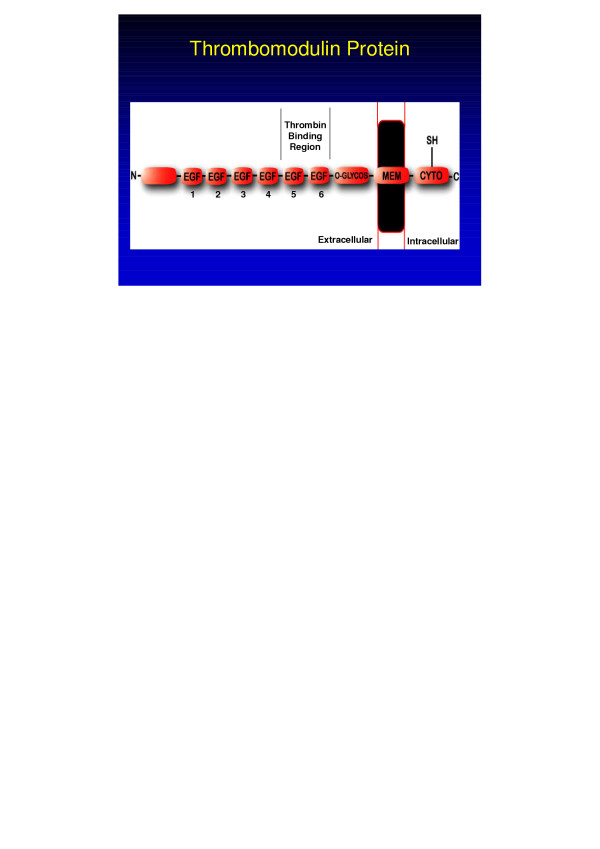
Thrombomodulin protein

The thrombomodulin gene (*THBD*) maps to chromosome 20p11.2, contains a single exon and no introns, and spans 4 kb (OMIM 188040, UniGene NM_000361, Locus Link 7056). The thrombomodulin protein is expressed primarily on the luminal surface of vascular endothelial cells and consists of 557 amino acids (aa) (60,300 Dalton): an N-terminal lectin-like module (aa 1–154), a hydrophobic region (aa 155–222), six epidermal growth factor (EGF)-like modules (aa 223–462), a serine and threonine rich region (aa 463–497), a single transmembrane segment (aa 498–521), and a short cytoplasmic tail (aa 522–557) [6]. A single nucleotide polymorphism (C→T) at position +1418 (C1418T) encodes for an aa change from alanine to valine at protein position 455 (Ala455Val) [7]. The location of this aa variation corresponds to the sixth EGF region of the thrombomodulin protein as seen in Figure 2. This location has been shown to be responsible for the high-affinity binding of thrombin and for the suspension of thrombin at a specific position above the endothelial surface in relation to other cofactors, thereby producing optimal protein C activation by thrombin [2,8].

A few studies have shown that the *THBD *Ala455Val polymorphism is associated with ischemic heart disease [9,10], but we know of no prior reports examining this polymorphism's association with stroke. Using data from the Stroke Prevention in Young Women Study [11], we sought to determine the association between the *THBD *Ala455Val polymorphism and the occurrence of ischemic stroke in young women. In addition, because cardioembolic stroke has a lesser degree of familial aggregation [12], we performed a secondary analysis excluding cases with cardioembolic etiologies.

## Methods

The Stroke Prevention in Young Women Study (SPYW) is a population-based case-control study that was initiated to examine risk factors for ischemic stroke in young women. In that study the term "population-based" means that cases and their comparison group were identified from the same defined population. The study area included all of Maryland (except the far Western panhandle), Washington DC, and the southern portions of both Pennsylvania and Delaware. Cases were female patients 15 to 44 years of age with a first cerebral infarction as identified by discharge surveillance at 59 regional hospitals and through direct referral by regional neurologists. The methods for discharge surveillance, chart abstraction, and case adjudication have been described previously [11,13,14]. The adjudication of stroke cases was performed blinded to genetic information. Stroke cases were classified as having a probable, possible or undetermined etiology as per prior description [13,14]. Control subjects were women without a history of stroke. They were identified by random digit dialing and were frequency matched to the cases by age and geographic region of residence. The original SPYW study consisted of 227 cases and 342 controls. DNA samples were available for a subset of this population consisting of 141 cases and 210 controls.

We performed *THBD *genotyping at the Ala455Val polymorphism for 141 cases and 210 control subjects. This included all case and control samples that were available at that time. Genotyping was performed blinded to case-control status. Genomic DNA was extracted from stored peripheral blood lymphocytes by using standard protocols (Gentra Systems, Minneapolis, MN). The *THBD *Ala455Val polymorphism was determined by pyrosequencing. The single-nucleotide polymorphism region of the gene was amplified by polymerase chain reaction (PCR) with the use of published primers [10] except that we labeled the reverse primer with biotin. PCR was performed in 40 μl reactions containing 40 ng of genomic DNA, 15 pmol each of forward and reverse primer, 1.5 U of Amplitaq (Applied Biosystems, Foster City, CA) and MasterAmp PCR PreMix D (Epicenter, Madison, WI). The resulting biotinylated PCR product was bound to streptavidin-coated Sepharose HP beads (Amersham Pharmacia Biotech, Uppsala, Sweden) and the product was denatured according to the manufacturer's protocol (PSQ 96 Sample Preparation Kit, Pyrosequencing AB, Uppsala, Sweden). Following denaturation, an internal sequencing primer (5'-CGACTCGGC CCT T-3') was annealed to the bound single-stranded DNA. We used an automated pyrosequencing instrument (PSQ96, Pyrosequencing AB, Uppsala, Sweden) to perform the genotyping [15,16]. The reactions were performed at 28°C and contained the bound single-stranded DNA with annealed sequencing primer, enzymes (DNA polymerase, apyrase, luciferase, and activating transcription factor sulfurylase), nucleotides (dTTP, dGTP, dCTP, or dATPαS), and substrate (luciferin) supplied by the manufacturer. We monitored continuously the output from the charge-coupled device as a pyrogram, and we analyzed manually the results from the completed sequencing reactions by visually inspecting each program. The validity of the method was confirmed by fluorescent dye terminator sequencing of a subset of samples using standard protocols on an ABI 3100 genetic analyzer (Applied Biosystems, Foster City, CA).

We assessed the following potential confounders of the association between the alleles of the *THBD *Ala455Val polymorphism and stroke: age, race, current cigarette smoking, hypertension, diabetes mellitus, history of angina or myocardial infarction (angina/MI), use of oral contraceptive pills (OCP) or hormone replacement therapy (HRT), sickle cell disease, and sickle cell trait. Age, race, current cigarette smoking status, use of OCP or HRT was determined by subject reports (or proxy report, if a participant was unable to answer). Hypertension and diabetes mellitus, sickle cell disease or sickle cell trait were determined by asking study participants (or a proxy) if a physician had ever told them that they had the condition.

We compared means by t tests and proportions by χ2 tests. The probability values presented are based on two-sided tests. Because of the low frequency of the V455 allele, we compared the frequency of the combined AV/VV genotype between cases and controls. Adjusted odds ratios derived from logistic regression were used to determine whether the presence of the Ala455Val test allele was associated with an increased risk for stroke after differences in age, race, current cigarette smoking, hypertension, and diabetes mellitus were controlled for. Additional analyses included: 1). adding ischemic heart disease (angina/MI) into the logistic regression model; 2). evaluation for interactions between genotype and OCP/HRT 3). an analysis excluding sickle cell trait, and 4). an analysis excluding cardioembolic strokes.

## Results

### Subject characteristics

Characteristics by case-control status are described in Table 1. The mean age of the cases (i.e., women with a first cerebral infarction) was 35.5 years and the mean age of control subjects was 36.1 years. Cases were more likely than control subjects to be black (44.0% versus 34.8%, p = 0.12), and were significantly more likely to currently smoke cigarettes (p < 0.001), to have hypertension (p < 0.01), diabetes (p < 0.001) and history of angina/MI (p < 0.001). No study subjects reported sickle cell disease, however 6 cases and 5 controls reported sickle cell trait (non-significant difference). Twenty cases and 34 controls reported use of oral contraceptive pills (OCP) or hormone replacement therapy (non-significant difference).

**Table 1:** Characteristics, by case-control status

	Case (N = 141)	Control (N = 210)	p-value
Mean age (years)	35.5	36.1	.31
Black (%)	44.0	34.8	.12
Current Smokers (%)	45.4	26.7	<.001
Hypertension (%)	27.7	13.3	<.01
Diabetes mellitus (%)	13.5	3.3	<.001
Angina/MI (%)	14.9	4.3	<.001

### Genotype and vascular risk factor distributions

The distribution of genotypes was in Hardy Weinberg equilibrium for the pooled set of cases and controls, both in total and by race.

Among control subjects, the prevalence of the AA genotype was 81% (59/73) for blacks and 68% (93/137) for whites. The relationship between the Ala455Val genotypes and selected stroke risk factors in control subjects is summarized in Table 2. Blacks were significantly more likely to have the AA genotype than the AV and VV genotypes combined (38.8% vs. 24.1%, p < 0.05). In contrast, there were no significant differences in prevalence of hypertension, diabetes, angina/MI, or sickle cell trait between carriers and non-carriers of the V allele, nor did the frequency of cigarette smoking or OCP/HRT use differ significantly between the two groups.

**Table 2:** Characteristics among control subjects, by thrombomodulin genotype status

	AA (n= 152)	AV/VV (n= 58)	p-value
Mean age (years)	36.5	34.9	0.17
Black (%)	38.8	24.1	<.05
Current Smokers (%)	26.3	27.6	0.86
Hypertension (%)	11.2	19.0	0.18
Diabetes Mellitus (%)	4.0	1.7	0.34
Angina/MI (%)	2.4	1.9	0.26

### Genotype risk

Table 3 shows the association of the AA genotype with stroke, stratified by race and other vascular risk factors. The association between the AA genotype and stroke was 2.7 (95% CI 0.9–8.0) among blacks and 1.6 (95% CI 0.8–3.2) among whites. Since logistic regression analysis did not show a significant interaction by race (i.e., the effect of the AA genotype did not differ significantly between blacks and whites), subsequent analyses were conducted on the combined sample. After adjustment for age, race, cigarette smoking, hypertension, and diabetes, the AA genotype was found to be significantly associated with stroke compared with the AV and VV genotypes (OR 1.9, 95% CI 1.1–3.3).

**Table 3:** Frequency of the *THBD *Ala455Val AA genotype in cases and controls (proportion with AA genotype in parentheses) as stratified by race and other stroke risk factors; with associated crude and adjusted odds ratios

Risk Factor	Percentage of cases with the AA genotype (proportion)	Percentage of Controls with the AA genotype (proportion)	Crude OR ^ (95% CI)	Adjusted OR*^ (95% CI)
White	79% (62/79)	68% (93/137)	1.7 (0.9–3.3)	1.6 (0.8–3.2)
Black	87% (54/62)	81% (59/73)	1.8 (0.4–7.9)	2.7 (0.9–8.0)
Current smoking	84% (54/64)	71% (40/56)	2.2 (0.9–5.3)	3.0 (1.1–7.8)
No current smoking	81% (62/77)	73% (112/154)	1.6 (0.8–3.0)	1.5 (0.7–2.9)
Hypertension	85% (33/39)	61% (17/28)	3.6 (1.1–11.3)	5.7 (1.4–22.6)
No hypertension	81% (83/102)	74% (135/182)	1.5 (0.8–2.8)	1.6 (0.8–3.0)
Diabetes**	84% (16/19)	86% (6/7)	Not performed	Not performed
No Diabetes	82% (100/122)	72% (146/203)	1.8 (1.0–3.1)	1.9 (1.1–3.4)
Angina/MI **	86% (18/21)	56% (5/9)	Not performed	Not performed
No Angina/MI	82% (98/120)	73% (147/201)	1.6 (0.9–2.9)	1.7 (.95–3.1)
Overall	82% (116/141)	72% (152/210)	1.8 (1.1–3.0)	1.9 (1.1–3.3)

* Each variable adjusted for age, race, smoking, hypertension, and diabetes (less the stratified variable). Overall model and Angina/MI adjusted for age, race, smoking, hypertension, and diabetes. Including Angina/MI in adjusted overall model demonstrated no change in association (OR = 1.9 95% CI = 1.1–3.3).

** Insufficient sample size to perform diabetic or angina/MI analyses.

^ The combined AV and VV genotypes within each strata serve as the reference group in all analyses, with the crude OR and adjusted OR assigned a reference value of 1.0.

The strength of association between the AA genotype and stroke remained unchanged including history of angina or myocardial infarction in the logistic regression model (OR 1.9, 95% CI 1.1–3.3). Neither OCP/HRT use, nor sickle cell trait demonstrated an interaction with genotype and additional adjustment for these factors did not alter the association between the AA genotype and stroke.

### Stroke subtype

Among the 141 stroke patients, 70 (50%) had a least 1 probable cause, 30 (21%) had no probable cause but a least one possible cause, and 41 (29%) were indeterminate. Table 4 shows the distribution of probable and possible causes. "Other determined causes" of stroke included hematologic disorders, nonatherosclerotic vasculopathy (eg, vasculitis and dissection), migraine, drug abuse and stroke associated with oral contraceptive or exogenous estrogen use.

**Table 4:** Etiologies among cases with a probable or possible cause of stroke

	Probable Causes^1 ^(n = 70)	Possible Causes^2 ^(n = 30)
Large-artery autherosclerosis	9	8
Cardioembolism*	16	14
Lacune	7	3
Other determined cause**	38	5

^1 ^One patient had 2 probable causes, but only 1 cause is listed according to the following hierarchy: large-artery atherosclerosis > cardioembolism > lacune> other determined cause.

^2 ^Most patients had multiple possible causes, but only 1 cause is listed per patient according to the same hierarchy as for probable causes.

* Note one probable case attributed to "other determined cause", also had possible cardioembolism as an etiology, this case was removed from the secondary analysis. A total of 31 cases were removed from the secondary analysis on the basis of either probable (n = 16) or possible (n = 15) cardioembolism as the stroke etiology.

** Other determined causes included: Probable = 38, (10 non-atherosclerotic vasculopathy, 13 hematologic, 4 migraine, 6 oral contraceptive or exogenous estrogen use, 5 other drug related). Possible = 5, (3 hematologic, 2 migraine).

A secondary analysis removing all probable (n = 16) or possible (n = 15) cardioembolic strokes was performed using the same adjusted model including age, race, smoking, hypertension, and diabetes. An increased association between non-cardioembolic stroke and the AA genotype was demonstrated (odds ratio 2.2, 95% CI 1.2–4.2).

## Discussion

In our study of the *THBD *Ala455Val polymorphism, the prevalence of the AA genotype among our control population was similar to that previously reported for the Atherosclerosis Risk in Communities (ARIC) Study population [10]. Our results indicate a positive association between the AA genotype and stroke among women aged 15 to 44 years. Furthermore, an increased association was demonstrated with the removal of all probable or possible cardioembolic strokes, a finding consistent with a recent meta-analysis demonstrating that cardioembolic stroke appears to have a smaller familial (or genetic) component that other subtypes of ischemic stroke [12]. Vascular risk factors were not significantly associated with specific genotypes in either analysis.

Several recent studies evaluating the *THBD *Ala455Val polymorphism and coronary artery disease (CAD) have yielded conflicting results. A Swedish case-control study found the alanine allele was associated with CAD [9]. In contrast, the American prospective ARIC study found the valine allele (AV plus VV) was associated with an increase in CAD risk in both blacks (OR 4.4, 95% CI 1.5–12.9) and whites (OR 1.4, 95% CI 0.9–2.1), although the association attained statistical significance only in blacks [10]. A British case-control study found no association at all between the *THBD *Ala455Val polymorphism and CAD [17]. Consistent with the Swedish results [9], we observed an association between the alanine allele at this locus and stroke onset at a young age. It is unclear whether the conflicting information regarding the *THBD *Ala455Val polymorphism, ours included, is due to population-stratification bias, a functionally neutral polymorphism that serves as a marker for a nearby functional mutation (linkage disequilibrium), or the true existence of different associations in the different study populations.

Population-stratification bias is due to confounding by population admixture [18]. An unidentified subpopulation can confound the association between a genotype and disease if the subpopulation is associated with the genotype under study and the risk of disease. Because our results indicate that blacks have a higher prevalence of the AA genotype and have an increased risk of early-onset stroke, the AA genotype might be a marker for African ancestry in general rather than a marker for increased stroke susceptibility.

The *THBD *Ala455Val locus may be in linkage disequilibrium with an unobserved "high-risk" susceptibility locus. Linkage disequilibrium is a function of the history of the population, and thus true associations can occur in one population and not another.

Our results are also consistent with a causal association between stroke and the *THBD *Ala455Val polymorphism, thereby defining a susceptibility locus for the disease. An important criterion for a true susceptibility locus is that the polymorphism is associated with a change in protein expression or function. The *THBD *Ala455Val polymorphism has not been associated with variation in soluble thrombomodulin concentrations [19], but soluble thrombomodulin levels do not necessarily indicate the functional status of thrombomodulin on the endothelial surface. The Ala455Val polymorphism resides within a critical region for thrombomodulin function, specifically within the sixth EGF region. Epidermal growth factor (EGF) regions 4, 5, and 6 within the thrombomodulin molecule (see Figure 1) appear to play critical roles in the activation of protein C by thrombin [2,8,20,21]. Furthermore, this contiguous EGF segment is the minimal functional fragment of the thrombomodulin cofactor that can switch the specificity of thrombin from a procoagulant to an anticoagulant enzyme [21,22]. Furthermore, two polymorphisms close to the Ala455Val polymorphism, Arg385Ser and Pro477Ser, have been shown to influence the expression and function of thrombomodulin in a tissue culture model [23].

## Conclusions

Thrombomodulin has not previously been examined as a candidate gene for stroke susceptibility. We found that among women aged 15 to 44 years, the AA genotype is more prevalent among blacks than whites and is associated with increased risk of early-onset ischemic stroke. Removing strokes potentially related to cardioembolic phenomena increased this association. Further studies are needed to determine whether this association is due to population stratification, linkage to a nearby functional polymorphism, or variation in thrombomodulin expression or function.

## Competing interest

The author(s) declare that they have no competing interests.

## Authors' contributions

All authors certify that they participated in the conceptual design of this work, the analysis of the data, and the writing of the manuscript to take public responsibility for it. All authors reviewed the final version of the manuscript and approve it for publication. J.W.C., S.J.K., B.D.M., M.A.W. and R.F.M. participated in the writing of the initial draft. M.G., K.K.S., W.H.G. and S.C.R. participated in the genotyping. J.W.C., S.J.K., B.D.M., and L.J.R. participated in the data analysis. All authors provided critiques of the final manuscript.

## Funding acknowledgments

Dr. Cole's effort on this project was supported in part by an American Academy of Neurology Clinical Research Training Fellowship, by the National Institutes of Health Research Training in the Epidemiology of Aging (Grant T32-AG00262-04), and by the Department of Veterans Affairs, Baltimore, Office of Research and Development, Medical Research Service, and Stroke Research Enhancement Award Program. Dr. Kittner was supported in part by the Department of Veterans Affairs, Baltimore, Office of Research and Development, Medical Research Service, Geriatrics Research, Education and Clinical Center, and Stroke Research Enhancement Award Program; a Cooperative Agreement with the Division of Adult and Community Health, Centers for Disease Control and Prevention; the National Institute of Neurological Disorders and Stroke and the NIH Office of Research on Women's Health; the National Institute on Aging Pepper Center Grant P60 12583; and the University of Maryland General Clinical Research Center (Grant M01 RR 165001), General Clinical Research Centers Program, National Center for Research Resources, NIH.

## Pre-publication history

The pre-publication history for this paper can be accessed here:


